# Early Positional Changes and Aesthetic Outcomes of Implant‑Supported Single Crowns in the Anterior Maxilla: A Cohort Study

**DOI:** 10.1002/cre2.70498

**Published:** 2026-10-29

**Authors:** Cecilia Turborn, Nicole Winitsky, Mattias Pettersson

**Affiliations:** ^1^ Department of Prosthodontics, Public Dental Health Service Region Västerbotten Umeå Västerbotten County Sweden; ^2^ Private Practice, Birka Tandhälsa Stockholm Stockholm County Sweden; ^3^ Centre of Dental Medicine University of Zürich Zürich Canton of Zürich Switzerland; ^4^ Division of Prosthetic Dentistry, Department of Odontology, Faculty of Medicine Umeå University Umeå Västerbotten County Sweden

**Keywords:** dental, dental Implants, dental impression technique, esthetics, imaging, three‐dimensional

## Abstract

**Introduction:**

Single‐tooth replacement in the aesthetic zone remains clinically challenging. Implant‐supported single crowns (ISSCs) provide predictable long‐term outcomes, yet complications such as infraposition, along with time‐dependent aesthetic changes, may still occur over time.

**Objectives:**

To evaluate early infraposition of ISSCs in the anterior maxilla of adult patients and assess aesthetic outcomes.

**Materials and Methods:**

Twenty‐five patients (median age 23 years) with ISSCs were followed for a median of 1.2 years. Positional changes were measured using TRIOS 3 intraoral scanner and TRIOS Patient Monitoring software (3Shape A/S, Copenhagen, Denmark). Aesthetic outcomes were assessed through Patient‐Reported Outcomes and Clinician‐Rated Outcomes, focusing on pink and white aesthetics via visual analogue scales. Overall satisfaction was recorded using a four‐level ordinal scale.

**Results:**

Median vertical movement of 0.16 mm (*IQR* = 0.19 mm; range = 0–1 mm; *Z* = −4.29; *p* < 0.001) while a median bucco‐palatal movement of 0.04 mm (*IQR* = 0.15 mm; range = −0.10–0.68 mm; *Z* = −2.44; *p* = 0.015) was found. Patients consistently rated aesthetic outcomes higher than clinicians, with statistically significant differences across all parameters and time points.

**Conclusions:**

ISSCs in the anterior maxilla exhibited small but statistically significant positional changes within 1 year after crown delivery. Patients rated the aesthetic outcome more favorably than clinicians. The clinical significance of these early changes remains uncertain and warrants further longitudinal investigation.

**Trial Registration:**

Not applicable due to observational study design.

## 1 Introduction

Replacing a single missing tooth in the anterior maxilla remains a clinical challenge due to the high aesthetic demands of this region. Placement of a dental implant followed by an implant‐supported single crown (ISSC) has long been considered the state‐of‐the‐art solution when clinical conditions permit (Bawa et al. 2023; Christensen 2008). While implant survival rates are excellent, exceeding 95% over decades (Barkarmo and Kowar 2025; Gotfredsen 2012; Winitsky et al. 2018; Pjetursson et al. 2012) survival of the ISSC tends to decline over time (Barkarmo and Kowar 2025; Gotfredsen 2012; Winitsky et al. 2018; E. Jung et al. 2012). In addition, complications are relatively common, with reported rates of more than 50% after 14–20 years (Winitsky et al. 2018). These complications may require corrective interventions such as restoration remake, soft tissue management, or orthodontic adjustments, thereby increasing treatment complexity, cost, and chair time, and potentially negatively affecting patient satisfaction.

Infraposition is a previously described complication (Andersson et al. 2013; Bernard et al. 2004; Nilsson et al. 2019; Schwartz‐Arad and Bichacho 2015; Thilander et al. 1999; Winitsky et al. 2021) that results from the ankylotic nature of dental implants, which prevents them from adapting to continuous craniofacial development and natural tooth eruption (Thilander et al. 1999; Forsberg et al. 1991; Thilander et al. 1992). As adjacent teeth continue their physiological eruption, the implant and its crown unit remain static, resulting in vertical discrepancies and, in some cases, buccal tilting and the loss of interproximal contacts (Winitsky et al. 2021; Byun et al. 2015; Thilander et al. 1994). The risk is particularly pronounced when implants are placed before skeletal growth is complete and therefore placement is frequently postponed until growth cessation (Thilander 1992, 1994, 2001). However, even in adults, subtle facial skeletal changes and ongoing tooth eruption can maintain the risk of infraposition throughout life (Forsberg et al. 1991; Al‐Taai et al. 2022).

Several aetiological factors have been associated with infraposition of ISSCs, including age, growth patterns, and facial characteristics such as Lower Anterior Face Hight (LAFH), sex, and the aetiology of missing teeth (Andersson et al. 2013; Bernard et al. 2004; Nilsson et al. 2019; Schwartz‐Arad and Bichacho 2015; Winitsky et al. 2021; Jemt et al. 2007; Brahem et al. 2017; Polymeri et al. 2020). However, the evidence remains inconsistent, and predictive models are still underdeveloped (Klinge et al. 2021; Papageorgiou et al. 2018). Moreover, most available data derive from studies with extended follow‐up periods (Andersson et al. 2013; Bernard et al. 2004; Schwartz‐Arad and Bichacho 2015; Thilander et al. 1999; Winitsky et al. 2021), leaving a gap in knowledge regarding early changes.

Recent advances in digital dentistry offer promising tools to address this gap. Intraoral scanners combined with monitoring software enable the capture of highly accurate digital models and facilitate longitudinal assessments of tooth position with minimal error (Michelinakis et al. 2020; Nedelcu et al. 2018a; Osnes et al. 2020). These technologies allow measurement of vertical and bucco‐palatal changes without relying on traditional methods, such as radiographs or plaster casts, which are more prone to variability (Nedelcu et al. 2018b). Integrating digital workflows into clinical research may therefore enhance the reliability of positional assessments, standardize follow‐up protocols, and support earlier detection of clinically relevant changes. Such approaches could also inform evidence‐based timing of definitive restorations, and potentially reduce overtreatment and improve cost‐effectiveness (Dimitrova 2025).

In addition, regulatory frameworks emphasize the structured collection of both Patient‐Reported Outcomes (PROs) and Clinician‐Rated Outcomes (ClinROs) in the evaluation of clinical success (BEST 2016). Research on the aesthetic outcomes of ISSCs consistently reports a discrepancy between PROs and ClinROs, with patients generally rating aesthetics more favorably than clinicians (Winitsky et al. 2021; Altay et al. 2019; Sadilina et al. 2025). Incorporating patient perspectives, even in cases involving minor positional changes, may contribute to a more comprehensive assessment of treatment outcomes and support evidence‐based follow‐up protocols.

The primary objective of this study was to investigate the frequency and extent of infraposition of ISSCs relative to adjacent teeth in the anterior maxilla during the first year after crown delivery and to explore potential contributing factors. The secondary objective was to evaluate aesthetic outcomes at baseline (BL) and 1‐year follow‐up (1Y FU) using both PROs and ClinROs. The null hypothesis states that no positional changes of adjacent teeth relative to the ISSCs would occur during the first year after crown delivery, regardless of patient‐ or treatment‐related factors.

## 2 Materials and Methods

This cohort study included 25 patients treated with ISSCs in the anterior maxilla between 2020 and 2024. The study was conducted in accordance with the principles outlined in the Helsinki Declaration (World Medical Association 2025). Patients were recruited from the Department of Prosthodontics at the Public Dental Health Service, Region Västerbotten, Umeå, Sweden, and from the Department of Prosthodontics at Folktandvården Eastmaninstitutet, Folktandvården Stockholms län AB, Stockholm, Sweden. Ethical approval was obtained from the Swedish Ethical Review Authority (Dnr 2021‐00133, with additional approval Dnr 2022‐02455‐02). In addition, an earlier approval from the Regional Ethical Committee in Stockholm (Dnr 2018/1677‐31/1) covers part of the material collected at Folktandvården Eastmaninstitutet, Stockholm. The study adheres to the STROBE guidelines (Cuschieri 2019). The authors used Microsoft Copilot for language refinement in the manuscript. All AI‑assisted content was reviewed and verified by the authors, who take full responsibility for the final version.

### 2.1 Participants and Study Variables

Patients over 18 years of age who had received an ISSC in the anterior maxilla (canine to canine) were eligible for inclusion. Ten patients from the Department of Prosthodontics, Public Dental Health Service, Region Västerbotten, Umeå, were included at the time of ISSC placement. An additional 15 patients from the Department of Prosthodontics, Folktandvården Eastmaninstitutet, Stockholm, were included at the 1Y FU. Although the Stockholm cohort entered the study at the 1Y FU, the BL data collected were comparable to those from the Umeå cohort, ensuring consistency across centers. All participants provided written informed consent.

The movement of the tooth mesial to the implant was monitored, and aesthetic outcomes were evaluated. In addition to these primary assessments, clinical variables were recorded at BL and at the 1Y FU, including sex, age at implant placement, medical history and medications, LAFH, pre‑prosthetic orthodontic treatment, cause of tooth loss, smile‑line, and type of temporary restoration. Implant‑related variables comprised implant type (bone‑level/tissue‑level), manufacturer, augmentation procedures, and implant position. ISSC‑related variables included restoration type (screw‑retained/cement‑retained), use of a temporary ISSC, and occlusion/articulation on the ISSC. Measurement procedures for tooth movement, LAFH, aesthetic evaluation, and smile‑line classification are detailed in the subsequent subsections. All data were pseudonymized prior to analysis.

### 2.2 Measurement of Tooth Movement

Intraoral scans captured at BL and at 1Y FU using TRIOS 3 intraoral scanners (3Shape A/S, Copenhagen, Denmark) were automatically aligned using the TRIOS Patient Monitoring software version 1.7.9.1. Measurements were taken in both vertical and bucco‐palatal directions (Figure 1) and carried out on the tooth mesial to the ISSC, as well as on the ISSC. The ISSC was assumed to be a stable reference point throughout the analysis. Each measurement was repeated three times, and the average value was calculated. Positional changes were then calculated by using the average values in relation to the stable ISSC reference. A positive value indicated that the mesial adjacent tooth in the 1Y FU model was positioned more inferiorly in the vertical dimension, or more palatially in the bucco‐palatal dimension, relative to the BL model.

**Figure 1:**
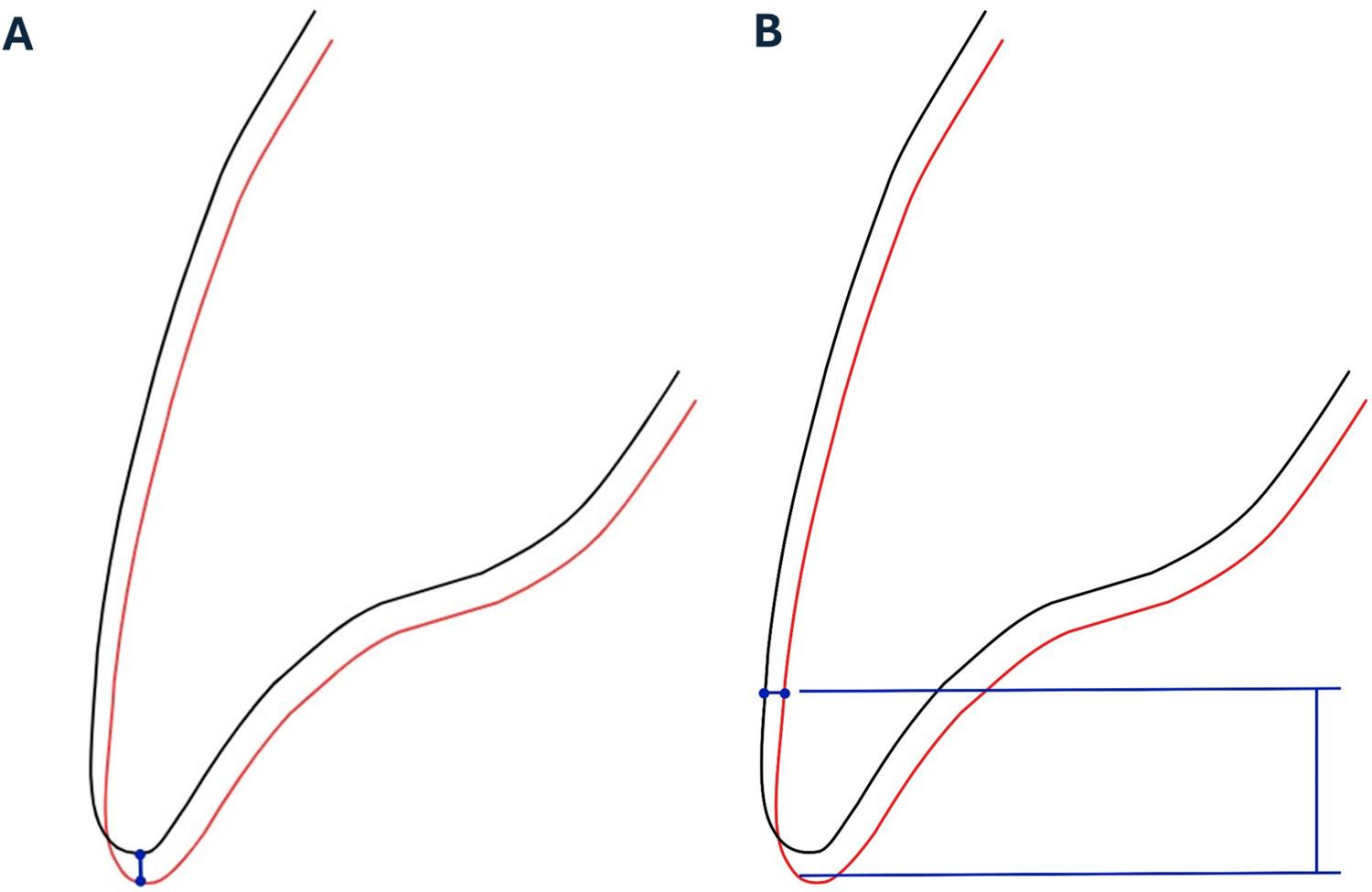
Schematic illustration of the measurement procedure. (A) Vertical measurements were performed on the aligned scans, perpendicular to the most inferior position of the incisal edge in the axial direction. (B) Bucco‐palatal measurements were made perpendicular to a point 2 mm in the axial direction from the buccal inclination of the incisal edge. Measurements were taken from the buccal surface of the BL model to the 1Y FU model.

### 2.3 Measurement Error Analysis

To address potential measurement bias, control measurements were conducted to evaluate the margin of error in scan alignment using the Trios Patient Monitoring software. A total of 16 scans were performed on a dentate maxilla with 14 teeth in the same individual on the same day. Half of the scans were performed from right to left, and the other half from left to right, with both sets starting with the most posterior tooth. The scans were then divided into right and left groups accordingly.

A model for comparison was randomly selected for each group, and the remaining models within the same group were aligned one by one. Measurements were carried out on the aligned models in the same manner as previously described and illustrated in Figure 1. The tooth positions for measurements in the maxilla were the most posterior teeth, the canines, and the central incisors. Using the registered values for each tooth position, a median margin of error for the measurement method in both the vertical and bucco‐palatal directions was calculated.

### 2.4 Measurement of LAFH

LAFH was clinically measured using a metal ruler. The measurement was taken from a horizontal plane beneath the chin, oriented parallel to the pupillary line, to the base of the nose (Figure 2). Contacts in occlusion, laterotrusion, and protrusion of the ISSCs were assessed using a doubled‐over red occlusion foil (12 µm) (Coltene/Whaledent, Altstätten, Switzerland) and recorded as either “yes” or “no.”

**Figure 2:**
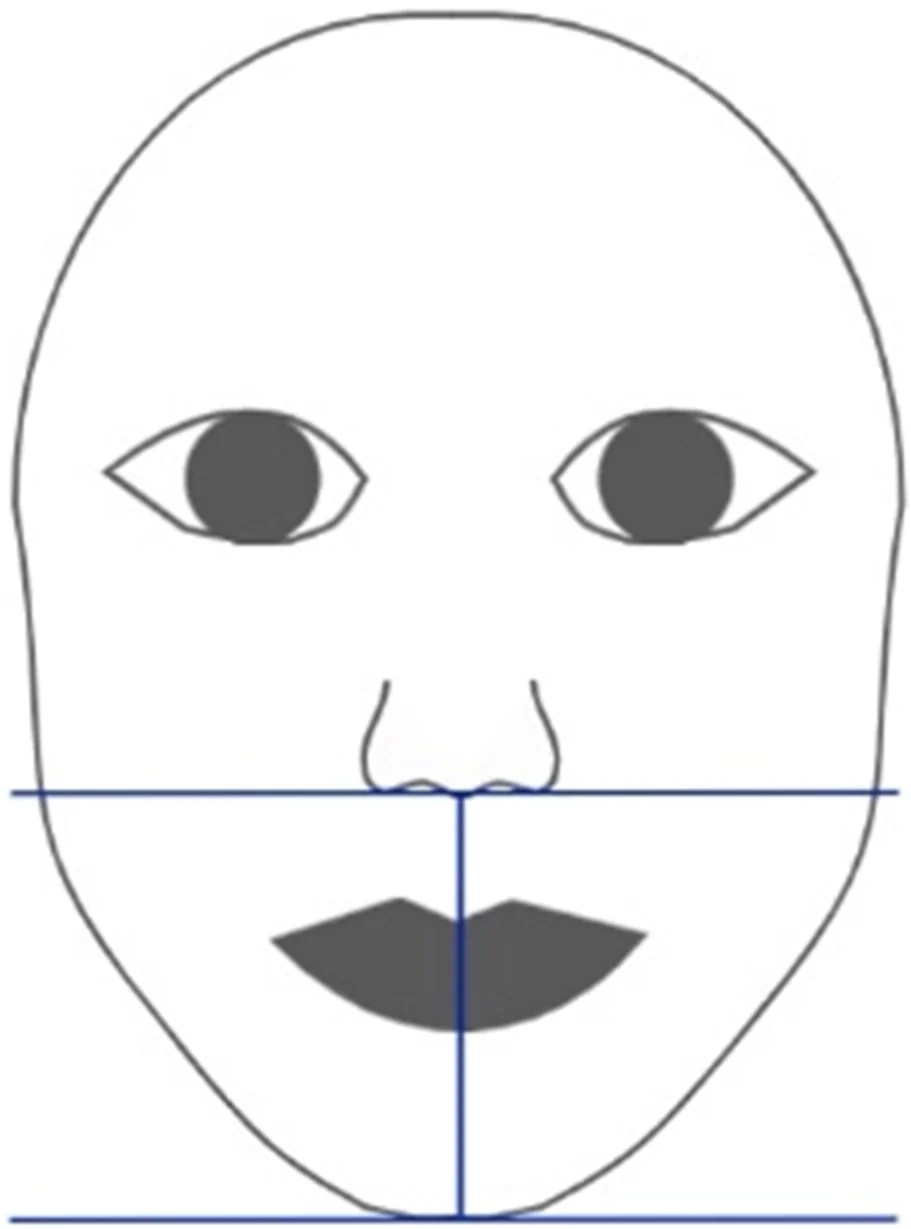
Schematic illustration of LAFH measurement.

### 2.5 Assessment of Aesthetic Outcome and Classification of Smile Line

Aesthetic outcomes were evaluated through both PROs and ClinROs. The assessments covered white aesthetics (PROwe, ClinROwe), pink aesthetics (PROpe, ClinROpe), and overall aesthetics (PROoverall, ClinROoverall). PROs were collected using a questionnaire, while ClinROs were performed by three clinicians (C.T., N.W., and M.P.) using standardized clinical photographs (Figure 3A,B).

**Figure 3:**
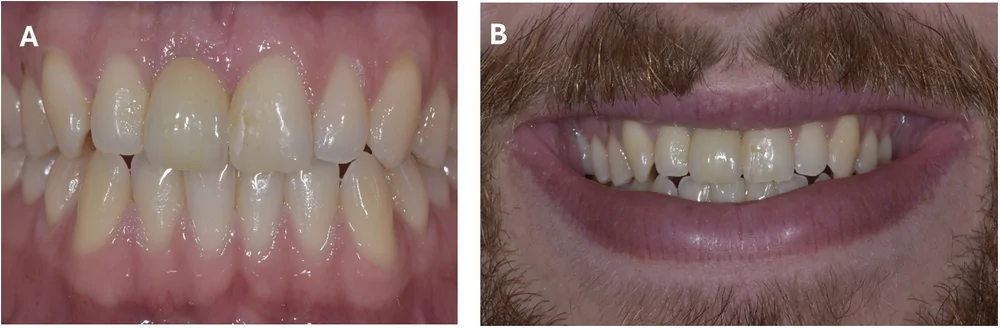
Assessment of ClinROs based on standardized clinical photographs with the photographer positioned at the same level as the patient. Lips retracted (A) for ClinROwe and ClinROpe and maximum smile photographs (B) for ClinROoverall.

For the patient‐reported assessments, PROwe was evaluated using the question: “How satisfied are you with the appearance of your implant‐supported tooth?,” whereas PROpe was evaluated using the question: “How satisfied are you with the appearance of your gum tissue?”. PROpe and PROwe were assessed using a visual analogue scale (VAS) of 100 mm (Chang et al. 1999), where rating was indicated by marking an “x” on a line ranging from 0 (“not satisfied at all”) to 100 (“fully satisfied”). PROoverall was assessed using the question: “How satisfied are you with the appearance of your implant‐supported tooth?,” recorded on a four‐level ordinal scale based on the framework by Stevens (Stevens 1946), defined as follows: 1 = “not satisfied at all,” 2 = “partially dissatisfied,” 3 = “partially satisfied,” and 4 = “fully satisfied,” the same ordinal scale was used for the ClinROoverall assessment. All assessments were performed independently by the patient and each clinician.

The photographs used for the evaluation were taken using a digital single‐lens reflex camera equipped with a macro flash. Camera settings included a shutter speed of 1/100 s and an aperture of f/28‐32 (Figure 3A), and f/20 (Figure 3B). All clinical photographs were compiled into a numbered list. The evaluation began with a randomly selected image, followed by the remaining images in descending order. Three independent evaluations were performed by each clinician on separate days, and an average value was calculated and used in the analysis.

Classification of smile line was performed by a single clinician (CT) according to Figure 4, following a method previously described (Winitsky 2023).

**Figure 4:**
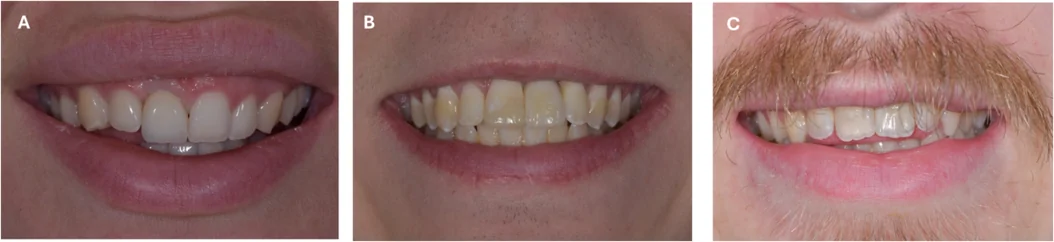
Smile line was determined as high (A); if mucosa was visible cervical to the ISSC, medium (B); if parts of the papillae region were visible surrounding the ISSC, and low (C); if no mucosa was visible.

### 2.6 Statistical Methods

Descriptive statistics were used to summarize the demographic and clinical characteristics of the study population and the ISSCs. Tooth position and VAS scores were treated as continuous variables, whereas overall aesthetic satisfaction ratings were considered as ordinal variables. The planned study size was determined based on effect estimates from a previous study (Winitsky et al. 2021). To detect a mean positional change of 1.0 mm with an assumed standard deviation of 0.5 mm, a sample size of approximately 16 participants per group (men/women) was required to achieve 80% statistical power at a two‑sided significance level of 0.05. Despite this target, recruitment was limited, and the final study cohort consisted of 25 patients. Analyses were based on all available data for each variable, and patients with missing values were excluded from analyses involving those variables. The number of observations and the proportion of missing data are reported in the respective tables.

Data distribution was assessed by the test of skewness and kurtosis. Results are presented as medians with interquartile ranges (*IQR*s) and full ranges (minimum‐maximum), due to the non‐normal distributed data.

To evaluate changes in tooth position over the follow‐up period and assess agreement between PROs and ClinROs, Wilcoxon's signed‐rank tests were employed. For each comparison, the test statistic (*Z*) and corresponding *p*‐value were reported, with statistical significance defined as *p* < 0.05.

Relationships between positional changes in the vertical and bucco‐palatal directions, as well as correlations between PROs and ClinROs, were analyzed using Spearman's rank‐order correlation, with statistical significance defined as *p* < 0.05.

Associations between potential contributing factors for infraposition and movement of the adjacent tooth were assessed using Mann–Whitney *U* test for dichotomous variables and the Kruskal–Wallis test for variables with more than two categories. Statistical comparisons were not performed for variables with fewer than five observations in one or more categories due to insufficient sample size. A *p*‐value < 0.05 was considered statistically significant.

All statistical analyses were performed using SPSS version 25 (IBM Corp., Armonk, NY, USA).

## 3 Results

Analysis of normality showed that the data were not normally distributed. Measurement error analysis revealed a median margin of error of 0.04 mm (IQR: 0.02 mm, range: 0.02–0.06 mm) in the vertical direction and 0.03 mm (IQR: 0.01 mm, range: 0.02–0.04 mm) in the bucco‐palatal direction.

### 3.1 Patients and Study Variables

All 25 patients (17 men, 8 women) registered at BL were eligible for the 1Y FU. BL assessments were conducted at 26 days (*IQR*: 42.5 days; range 0–72 days) after crown delivery, and 1Y FU assessments at 1.2 years (*IQR*: 0.6 years; range: 0.9–1.9 years). The median age of the patients at implant surgery was 23 years (*IQR*: 7 years; range: 19–59 years), and at delivery of ISSC, 23 years (*IQR*: 7 years; range: 20–60 years). All patients received bone‐level implants and screw‐retained ISSCs. No diseases or medications known to influence continuous skeletal development were present in the cohort. Additional patient, implant, and ISSC characteristics are shown in Tables 1 and 2.

**Table 1:** Patient characteristics, presented as number and percentage [*n* (%)].

	Number (*n*)	Percentage (%)
Patients	25	
Sex		
Male/female	17/8	68/32
Cause of tooth loss		
Trauma	16	64
Agenesia	2	8
Delayed extraction due to previous pathology	7	28
Pre‐prosthetic orthodontic treatment	9	36
Temporary prosthesis	23	92
Removable partial denture (RPD)	5	20
Resin‐bonded bridge (RBB)	18	72
LAFH† (< 70 mm/≥ 70 mm)	10/14	40/56
Smile line (high/medium/low)	5/16/4	20/64/16

^†^ Missing data: LAFH (*n* = 1; 4%).

**Table 2:** Implant and ISSC characteristics, presented as number and percentage [*n* (%)].

	Number (*n*)	Percentage (%)
Implants		
Implant position		
(Central incisor/lateral incisor/canine)	23/2/0	92/8
Implant manufacturer		
(Nobel biocare/straumann/dentsply)	21/2/2	84/8/8
Bone augmentation (yes/no)	12/13	48/52
ISSC		
Temporary ISSC	2	8
Occlusion and articulation on ISSC at 1Y†		
Occlusion (yes/no)	4/19	17/83
Protrusion (yes/no)	6/17	26/74
Laterotrusion (yes/no)	4/19	22/78

^†^ Missing data: occlusion and articulation (*n* = 2; 8%).

### 3.2 Movement of Adjacent Tooth

The median positional change was 0.16 mm (*IQR* = 0.19 mm; range: 0.00–1.00 mm; *Z* = −4.29; *p* < 0.001) in the vertical direction and 0.04 mm (*IQR* = 0.15 mm; range: −0.10–0.68 mm; *Z* = −2.44; *p* = 0.015) in the bucco‐palatal direction. Since the positional change was statistically significant, the null hypothesis was rejected. A moderate positive correlation was found between vertical and bucco‐palatal positional changes (*ρ* = 0.47, *p* = 0.018). The distribution of movement data are presented in Figure 5.

**Figure 5:**
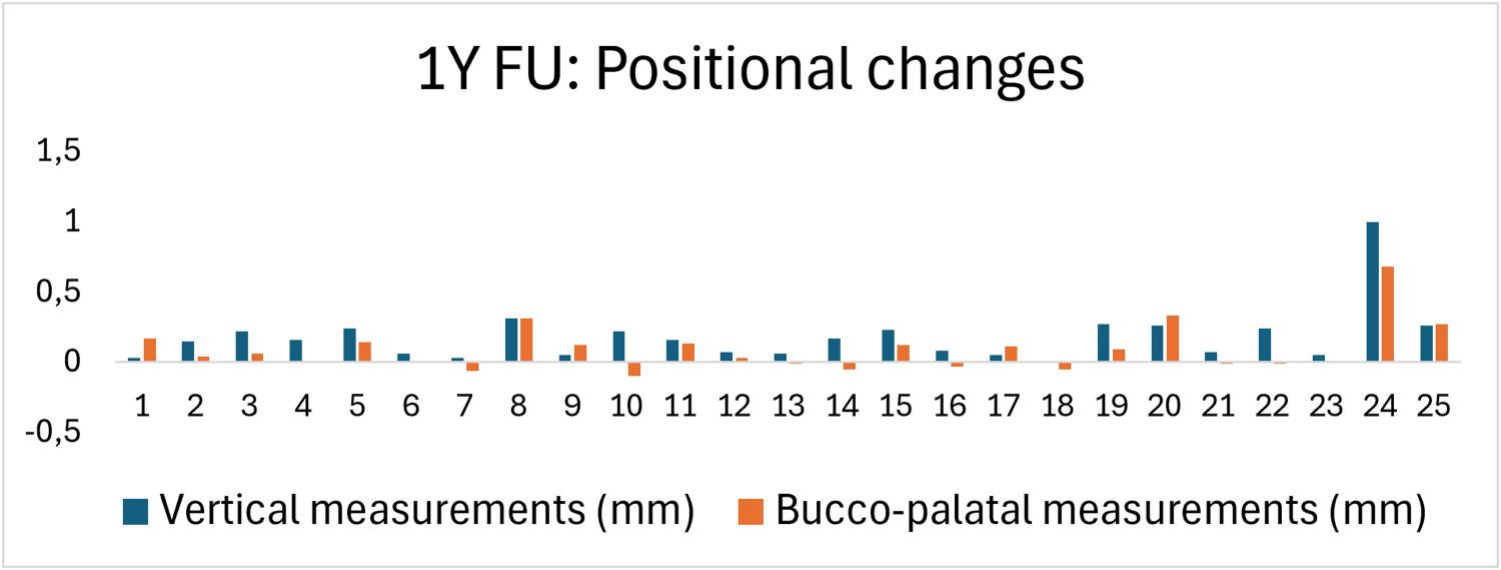
Distribution of positional changes of teeth mesially adjacent to ISSCs (mm) in vertical and bucco‐palatal directions for patients 1–25.

Table 3 presents vertical movement stratified by the investigated variables.

**Table 3:** Vertical movement of the adjacent tooth (mm) at 1Y FU, presented as median (IQR).

	*n*	Median	[IQR] range	*p*‐value
Patients	25	0.16	[0.19] 0.00–1.00	
Sex				0.43
Male	17	0.16	[0.18] 0.00–0.31	
Female	8	0.16	[0.19] 0.05–1.00	
Implant position				‡
Central incisor	23	0.15	[0.18] 0.00–0.31	
Lateral incisor	2	0.62	[–] 0.24–1.00	
Age at delivery of ISSC				0.26
< 30Y	20	0.17	[0.18] 0.00–1.00	
> 30Y	5	0.06	[0.12] 0.05–0.26	
Cause of tooth loss				‡
Trauma	16	0.17	[0.17] 0.03–0.31	
Agenesia	2	0.62	[−] 0.24–1.00	
Others	7	0.05	[0.02] 0.00–0.26	
Pre‐prosthetic orthodontic treatment				0.07
Yes	9	0.22	[0.16] 0.06–1.00	
No	16	0.07	[0.19] 0.00–0.27	
Temporary prosthesis				‡
RPD	5	0.07	[0.15] 0.00–0.27	
RBB	18	0.17	[0.18] 0.03–1.00	‡
None	2	0.15	[–] 0.05–0.24	
Temporary ISSC				
Yes	2	0.04	[–] 0.03–0.05	
No	23	0.16	[0.18] 0.00–1.00	
LAFH†				0.20
< 70 mm	10	0.21	[0.20] 0.05–1.00	
≥ 70 mm	14	0.12	[0.18] 0.00–0.31	

^†^ Missing data: LAFH (*n* = 1; 4%).

^‡^ Statistical comparisons were not performed for variables with < 5 observations.

### 3.3 Aesthetic Outcome

The PRO aesthetic outcomes at BL showed median PROwe, PROpe, and PROoverall scores of 96.9% (IQR = 7.2; range 86.3–100.0), 91.8% (IQR = 17.4; range 10.0–100.0), and 4.0 (IQR = 0.25; range 3.0–4.0), respectively. At the 1Y FU, the corresponding values were 96.9% (IQR = 15.4; range 64.7–100.0), 91.2% (IQR = 20.5; range 30.9–100.0), and 4.0 (IQR = 0.0; range 3.0–4.0), respectively.

The ClinRO aesthetic outcomes at BL showed median ClinROwe, ClinROpe, and ClinROoverall scores of 59.9% (IQR = 15.1; range 30.8–80.9), 51.3% (IQR = 16.2; range 15.8–77.1), and 2.8 (IQR = 0.8; range 1.6–3.9), respectively. At the 1Y FU, the corresponding values were 61.1% (IQR = 16.7; range 26.1–79.5), 58.0% (IQR = 18.4; range 28.3–76.8), and 2.8 (IQR = 0.7; range 1.6–3.0), respectively.

Aesthetic outcomes for pink and white parameters are shown in Figure 6, see Supporting Information S1: Appendices A1 and A2 for individual patient VAS scores. Statistically significant difference was found between PROs and ClinROs for both pink‐ and white aesthetics and for overall aesthetic evaluation, at both BL and 1Y FU. At BL, significant differences were observed between PROwe and ClinROwe (*Z* = −3.82, *p* < 0.001), between PROpe and ClinROpe (*Z* = −3.10, *p* < 0.002), and between PROoverall and ClinROoverall (*Z* = −3.10, *p* < 0.002). These differences remained significant at the 1Y FU: PROwe versus ClinROwe (*Z* = −4.20, *p* < 0.001), PROpe versus ClinROpe (*Z* = −3.69, *p* < 0.001), and PROoverall versus ClinROoverall (*Z* = −3.89, *p* < 0.001).

**Figure 6:**
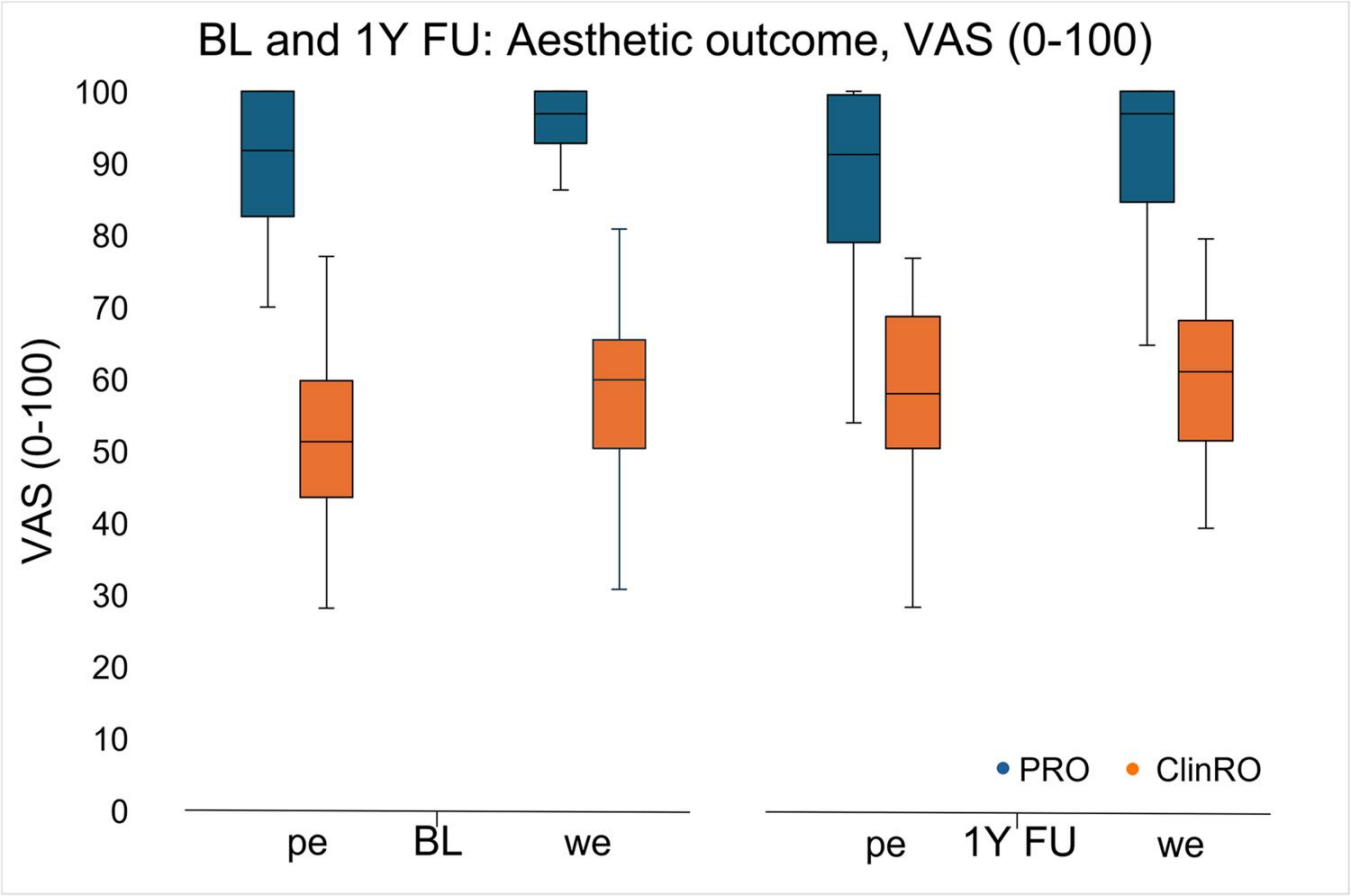
Boxplots showing PROpe, ClinROpe, PROwe, and ClinROwe at BL and at 1Y FU. The vertical axis represents VAS scores, boxes represent IQR, and horizontal lines indicate the median.

The overall aesthetic evaluations at the 1Y FU are illustrated in Figure 7. PROoverall at 1Y FU was rated as either “fully satisfied” or “partially satisfied,” 72% of the ClinROoverall was rated as “fully satisfied” or “partially satisfied.” At the 1Y FU, a statistically significant correlation was observed between PROoverall and PROwe (*ρ* = 0.61, *p* < 0.002), while no correlation was found with PROpe. For clinicians, both aesthetic scores were strongly correlated with ClinROoverall (ClinROwe: *ρ* = 0.68, *p* < 0.001; ClinROpe: *ρ* = 0.81, *p* < 0.001). No correlations were found between positional change and any of the assessed aesthetic parameters (WE, PE, or overall) in either group at the 1Y FU.

**Figure 7:**
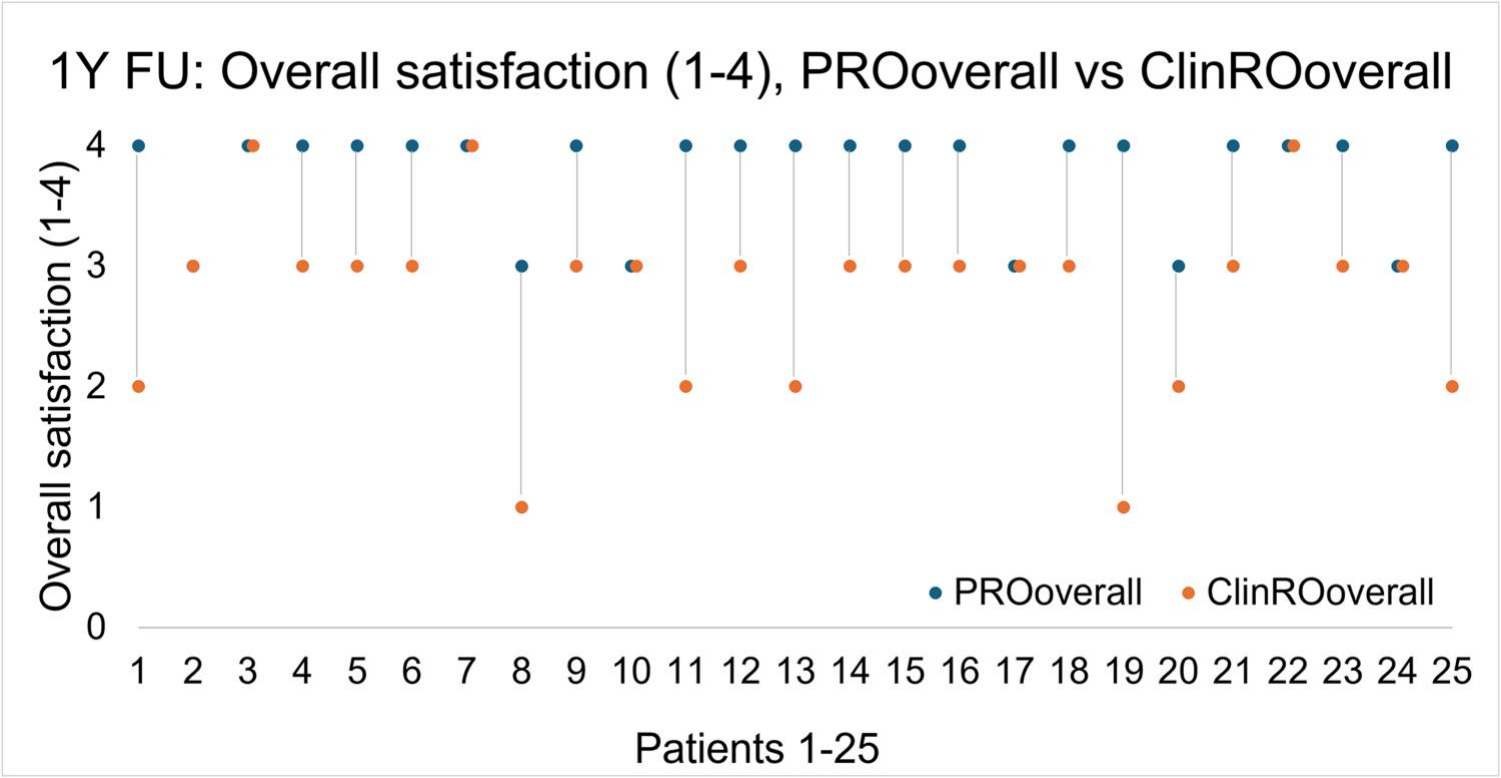
PROoverall, ClinROoverall at the 1Y FU shown per patient number 1–25. Higher scores indicate greater satisfaction (4 = “fully satisfied,” 1 = “not satisfied at all”). Data for PROoverall is missing for patient number 2. Patients 2, 6, 15, 19, and 21 presented with a high smile line.

## 4 Discussion

The primary findings of this cohort showed that early positional changes of adjacent teeth occurred within the first year following crown delivery, with vertical displacement being more pronounced than bucco‐palatal. Furthermore, patients reported significantly higher aesthetic satisfaction compared to clinicians. These primary findings were statistically significant.

The predominance of vertical movement and the positive correlation between greater vertical displacement and greater bucco‐palatal displacement observed in the present study are consistent with a previous report (Winitsky et al. 2021). From a clinical perspective, the observed vertical positional change is small and unlikely to be readily perceived by patients or to substantially influence their assessment of the aesthetic outcome. Although long‐term studies have shown that patient satisfaction generally remains high despite infraposition exceeding 1 mm, cases requiring crown replacement due to infraposition have also been reported. This suggests that the clinical consequences of infraposition may vary depending on the extent of the positional change and individual patient expectations (Andersson et al. 2013; Winitsky et al. 2021).

Although the median vertical positional change observed in the present study was only 0.16 mm, it may nevertheless be biologically relevant. The changes were detected within a relatively short period after crown delivery and exceeded both the annual rates reported in studies with longer follow‐up periods and the estimated average annual infraposition rate of 0.05 mm reported in a systematic review (Andersson et al. 2013; Winitsky et al. 2021; Polymeri et al. 2020; Papageorgiou et al. 2018; Sauvin et al. 2022). These findings suggest that positional changes may be more pronounced during the early period after crown delivery and subsequently stabilize over time. However, whether this pattern reflects the initial phase of the long‐term infraposition process remains unknown.

Causality cannot be established, and it remains unclear whether early and long‐term positional changes occur in the same individuals. Nevertheless, these findings may indicate a treatment‐related influence on early positional dynamics, suggesting factors beyond continuous jaw development and the natural eruption of adjacent teeth (Forsberg et al. 1991; Thilander 1992, 2001; Al‐Taai et al. 2022). One possible contributing factor could be immobilization of neighboring teeth by RBBs, which may explain the larger positional change observed in this group at the 1Y FU. In contrast, the smaller positional changes seen in the patients with RPDs may reflect the allowance for continued natural growth and eruption during the treatment period. Furthermore, the use of temporary ISSC restorations before definitive ISSC placement may allow adjacent teeth to align prior to ISSC delivery, which could help explain the reduced movement observed in this group.

Heterogeneity among contributing factors for infraposition has been stated in systematic reviews (Klinge et al. 2021; Papageorgiou et al. 2018). While some studies have found female sex to be a significant factor (Andersson et al. 2013; Jemt et al. 2007), others have not confirmed this association (Bernard et al. 2004; Nilsson et al. 2019; Winitsky et al. 2021; Brahem et al. 2017; Polymeri et al. 2020). In the present study, the degree of positional changes was similar regardless of sex. The younger patients (< 30 years) in this cohort presented with greater vertical discrepancies, which have been observed earlier (Schwartz‐Arad and Bichacho 2015), whereas other studies did not find such a relationship (Andersson et al. 2013; Bernard et al. 2004; Nilsson et al. 2019; Schwartz‐Arad and Bichacho 2015; Winitsky et al. 2021; Brahem et al. 2017). The greater positional changes observed in patients with agenesis and in those with implants placed in the lateral incisor region are in agreement with findings from a previous long‐term follow‐up study (Winitsky et al. 2021). Additional exploratory analyses also suggested greater positional changes among patients with a history of preprosthetic orthodontic treatment. Although none of these associations reached statistical significance, likely due to the limited sample size and corresponding limited statistical power, they may represent clinically relevant hypotheses for future research.

Consequently, the present findings should be regarded as hypothesis‐generating rather than confirmatory. Future longitudinal studies with larger cohorts are needed to determine whether treatment‐related factors, including orthodontic history and temporary prosthetic design, influence the development of early positional changes and their progression to clinically relevant infraposition.

The consistently reported higher satisfaction with the aesthetic outcome reported by the patients in this study supports previous evidence suggesting that patients are generally less critical of aesthetic deviations (Andersson et al. 2013; Bernard et al. 2004; Winitsky et al. 2021; Byun et al. 2015; Belser et al. 2009). This is well worth considering in the context of potential overtreatment in these complex cases, especially when patients’ aesthetic satisfaction remains high despite clinical deviations (Sadilina et al. 2025). Nevertheless, the pronounced difference between self‐assessments and professional evaluations underscores the importance of integrating both perspectives when determining aesthetic outcomes, especially in the aesthetic zone where subjective perception plays a decisive role.

The strong and statistically significant correlation found between PROoverall and PROwe suggests that patients place greater importance on the white than on the pink aesthetics. For clinicians, both aesthetic scores (ClinROwe, ClinROpe) were strongly correlated with overall satisfaction scores, suggesting that the trained professional eye values aesthetics more critically.

A strength of the present study is the use of digital methodology, which allows high precision through digital scanning and measurements. This was confirmed by the measurement error analysis, which confirms methodological reliability. In contrast to previous studies evaluating risk factors for infraposition, which employed varying measurement methods such as radiographs, clinical photographs, and digitally measured plaster (Andersson et al. 2013; Bernard et al. 2004; Nilsson et al. 2019; Schwartz‐Arad and Bichacho 2015; Thilander et al. 1999; Winitsky et al. 2021; Brahem et al. 2017; Polymeri et al. 2020), this study benefits from a continuous digital workflow. Despite these strengths, the study has limitations. The relatively small sample size affects the generalizability of the findings and reduces the statistical power for subgroup analyses. Measuring using Trios Patient Monitoring does not ensure that measurements are taken from a precise point from the BL model to the 1Y FU model. In addition, as measurements are taken at the incisal edge, an area known for wear, this represents a methodological limitation. Since traumatic tooth loss constituted the most common indication for treatment in the present cohort, trauma‐related factors may have influenced the observed positional changes. Detailed information regarding alveolar fractures and fracture fixation procedures was not available for all patients and could therefore not be included in the analyses. Another limitation lies in the objective aesthetic evaluation based on photographs. Although efforts were made to standardize the photographic documentation, differences in cameras and clinical environments limited the consistency of colors and light in the images.

The study material was collected at two dental specialist clinics, one in Umeå and one in Stockholm. Although both are referral centers, differences in regional patient characteristics and workflows may limit generalizability to other settings. Still, using two independent specialist clinics improves external validity compared with a single‐center study.

Clinically, the findings are relevant for monitoring and treatment planning to achieve stable short‑ and long‑term post‑treatment outcomes. Extended follow‑up studies with larger cohorts are needed to determine whether early changes stabilize or progress over time and to identify patient‑ or treatment‑related factors associated with more pronounced infraposition. Particular attention should be given to the potential influence of the temporary prosthesis, which may affect the development of early infraposition. Further work is required to refine aesthetic assessment methods so that they are both reliable and clinically meaningful.

Taken together, these findings support structured follow‑up protocols and informed treatment planning to optimize outcomes for patients receiving ISSCs.

## 5 Conclusion

Within 1 year after crown delivery, ISSCs in the anterior maxilla exhibited small but statistically significant positional changes. The magnitude of these changes was limited, and their long‐term clinical significance beyond the first year remains uncertain. Patients rated the aesthetic outcome more favorably than clinicians. Further longitudinal studies with larger cohorts and longer follow‐up periods are needed to determine whether these early positional changes progress to clinically relevant infraposition and to validate the present findings.

## Author Contributions

**Cecilia Turborn:** conceptualization, methodology, data collection, statistical analysis, and manuscript drafting. **Nicole Winitsky:** conceptualization, study design, clinical data acquisition, data interpretation, and critical revision of manuscript. **Mattias Pettersson:** conceptualization, study design, supervision, data interpretation, and manuscript review and editing.

## Ethics Statement

The study was approved by the Swedish Ethical Review Authority (Dnr 2021‐00133 and Dnr 2022‐02455‐02). An earlier approval from the Regional Ethical Committee in Stockholm (Dnr 2018/1677‐31/1) also applies.

## Consent

Written informed consent was obtained from all patients.

## Permission to Reproduce Material From Other Sources

Not applicable, no material from other copyrighted sources has been reproduced or adapted. All figures and tables are original and created by the authors.

## Conflicts of Interest

Nicole Winitsky lectures for, and receives personal fees, from Nobel Biocare Services AG, Zurich, Switzerland, outside the submitted work.

## Supporting information


Supporting File 1

Supporting File 2

## Data Availability

The data sets generated and/or analyzed during the current study are available from the corresponding author on reasonable request.
