# Supplementary material for: Early Positional Changes and Aesthetic Outcomes of Implant‑Supported Single Crowns in the Anterior Maxilla: A Cohort Study
Source: Clin Exp Dent Res. 2026 Oct 29;12(5):e70498. doi: 10.1002/cre2.70498 (PMC13719050; doi:10.1002/cre2.70498)
Supplement: Supplementary file 2 [file CRE2-12-e70498-s002.docx]

Appendix A1. 1Y FU VAS (0-100%), comparing PROpe and ClinROpe for patients 1-25. Data for PROpe is missing for patient number 2.

Appendix A2. 1Y FU VAS (0-100%), comparing PROwe and ClinROwe for patients 1-25. Data for PROwe is missing for patient number 2.
